# The Cost of Provisions in Institutions.—II

**Published:** 1904-04-16

**Authors:** 


### THE COST OF PROVISIONS IN INSTITUTIONS.—II.

By the Author of "Hospital Expenditure—The Commissariat."

(*Continued from page* 32.)

### THE SECRET OF ECONOMY.

The minimum of expenditure on provisions in small as well as large establishments can only be maintained by the observance of a threefold law of economy—economy of prices, economy of numbers, and economy of distribution. It is not generally understood that the first and the last of these should grow intertwined, and that economy of price, unsupported by economy of distribution is one of the most fatal delusions which ever destroyed the welfare of a household. In plain words, however low the price of provisions may be, they are not cheap unless they are of good quality. Bad food is wasted before preparation by the chopping and trimming necessitated in the kitchen; it is wasted in the carving, and it is wasted on the plates, where the hungry diners turn it wistfully over and let it lie.

Strange as it may seem, however, bad food is not usually the result of low prices. It is the result of dishonesty on the part of the contractor, carelessness on the part of the receiver, and incapacity on the part of the cook. An instance has recently come to our knowledge of rather flagrant underfeeding and bad meat in a nurses' home, where, from the exceptional demands made on the nursing staff for the service of the poor, a high standard of food is imperatively called for. An examination of the published accounts shows that the cost per head of the whole household, including servants, works out at no less than £20 a year for provisions—an average sufficient to provide every one with excellent food and leave a margin over for delicacies. The cause of this blot on an otherwise admirable institution can be traced to the want of a housekeeper, the lady superintendent being entirely absorbed by other duties. It is necessary to indicate this very clearly in discussing prices—that quality is quite as essential to economy as cheapness; but for the encouragement of the economist it may be affirmed that the two can by rigid care be made to walk hand in hand together, and that in no institution is to be found a higher standard of food than in some of those where the cost is lowest.

Some years ago an attempt was made to compare prices paid in London hospitals and Poor-law infirmaries for their main supplies, and the discovery was made that nothing like a standard price was ascertainable. The following prices per lb. were paid in London hospitals for legs of mutton: 3¼d., 4⅛d. (all-round price), 4¼d., 5d., 5¼d., 5½d., 5¾d. (all-round price), 5⅞d. (all-round price), 6½d., 7d. (all-round price), 7½d, 8d, 8½d. In the Poor-law infirmaries, also, every gradation of price from 3d. to 8d. per lb. was paid for the same article. And at the present day the same variations of price are to be met with.

A similar variation of price was found to exist in the supply of milk, the cost of which varied between 7d. and 11d. the imperial gallon.

The cost of potatoes afforded another illustration of the ignorance about prices prevalent in London hospitals, for only two institutions were found to agree in paying the same price, and the variation was from 2s. 9d. to 6s. 6d. per cwt. Reckoning that each inmate would consume about a hundredweight of potatoes in a year, such a difference in the price of this one insignificant article would mean an extra hundred pounds in even a moderate-sized hospital.

It is not very creditable to managers of institutions that they should appear to ignore the existence of regular market prices showing distinct seasonal fluctuations and subject to a general rise or fall when regarded through a series of years. It is much to be feared that those who order the supplies in many hospitals have grown accustomed to rely blindly on the contractor, and shirk the labour of themselves investigating through independent sources the cost of the commodities supplied, and the standard of quality which can be expected at the price quoted.

In a previous article we showed that a general increase, amounting to as much as £2 per occupied bed, had taken place in the rate of provisioning the hospitals. It is important to ascertain to what extent prices may be considered responsible for this general rate of increase in the cost of provisions.

We have before us a chart which shows the fluctuations in prices of some of the stock articles of hospital consumption during the last few years. It is part of a "Report on Wholesale and Retail Prices in the United Kingdom in 1902, with Comparative Statistical Tables for a Series of Years," and has been issued by the Board of Trade.

Starting with the year 1890, we find the price of beef falling steadily after that year until it reached its minimum in 1898, after which it rose till 1900, dropped slightly in 1901, and reached its maximum for the last 17 years in 1902. The price of mutton reached the minimum in 1893. In 1895 and 1900 respectively it touched its highest price for the last 10 years, but in 1901 it had again dropped; its fluctuations in price during this period were very slight, extending to but four points in all. Turning to the line which indicates the price of fish, we find a remarkable series of fluctuations extending over nearly 50 points. Taking the mean in the year 1891, we find a steady decline in prices of some 30 points, till the minimum is reached in 1896 (with a slight rise the previous year). From the year 1896 the rise to the maximum price nearly 50 points higher in 1899 was very sudden. From that point it sank to below the mean in 1901, but is now showing a slow upward tendency. Milk, which reached its maximum for the last 17 years in 1893-4, fell nearly 20 points to its minimum in 1898, and has shown a tendency since that year to a very slow rise of a few points. The fluctuations in the price of eggs follow very closely those of milk. The price of tea shows a steady decrease amounting to 60 points during the last 25 years, and that of coffee has declined 60 points since the year 1896. The price of potatoes is lower in 1901 than ever before, and that of bacon is but five points higher than the minimum, which is reached in 1898.

With the exception of beef, which was slightly higher in price in 1901 than in the other years indicated, there is nothing in the general trend of prices to account for the higher cost of provisions. But if the increased cost in the hospital commissariat cannot be traced to any general rise in prices, it is at any rate clear that difference in prices is at the bottom of much of the variation which is observable in the cost of provisions as between one institution and another.

A low proportion of "inmates other than patients" does much in reducing the average cost of board calculated on the basis of occupied beds. Rigid economy in distribution and restriction of waste does even more. But in these big establishments, if the prices are too high, by even a fraction, the efforts of the economist will fail to produce due effect on the bills, while distinctions in numbers will count for little.

The extent to which minute variations in price are forced into angry prominence by the size of the institution can be seen by considering how an extra farthing would affect the cost of some 100,000 lb. of meat. Can we honestly believe

ARMY MEDICAL LIBRARY

that every extra farthing on the way from 3d. to 8d. per lb. marks a gradation in the quality for which value in proportion is received?

The whole question of the meat supply is so complicated, and so closely involved with sharp practice, that it can never be properly solved for London institutions until a co-operative hospital meat store with model *abattoir* is established. But pending this solution of the difficulty much might be done by placing at the head of the commissariat department a skilled steward, of sufficient standing to obviate any risk of collusion with the contractor; he should be trained to distinguish at a glance good meat from bad, and English from foreign, and should be responsible for the supervision of all contracts, and for receiving and weighing all the supplies. Until there is some one in the institution whose business it is to know as much about the food supplies as the contractors (and the knowledge is not inaccessible), fancy prices will continue to be paid and the quality of the provisions will continue to be entirely dissociated from their cost.

We propose in succeeding articles to analyse the published accounts of a few typically well-administered London hospitals, with a view to placing at the service of hospital managers the experience of those who have made a special study of the commissariat department.

